# *Bifidobacterium longum subsp. longum* OLP-01 Supplementation during Endurance Running Training Improves Exercise Performance in Middle- and Long-Distance Runners: A Double-Blind Controlled Trial

**DOI:** 10.3390/nu12071972

**Published:** 2020-07-02

**Authors:** Che-Li Lin, Yi-Ju Hsu, Hsieh-Hsun Ho, Yung-Cheng Chang, Yi-Wei Kuo, Yao-Tsung Yeh, Shin-Yu Tsai, Ching-Wei Chen, Jui-Fen Chen, Chi-Chang Huang, Mon-Chien Lee

**Affiliations:** 1 Department of Orthopedic Surgery, Shuang Ho Hospital, Taipei Medical University, New Taipei City 23561, Taiwan; 11010@s.tmu.edu.tw; 2 Department of Orthopedics, School of Medicine, College of Medicine, Taipei Medical University, Taipei City 11031, Taiwan; 3 Graduate Institute of Sports Science, National Taiwan Sport University, Taoyuan City 33301, Taiwan; ruby780202@ntsu.edu.tw; 4 Glac Biotech Co., Ltd., Tainan City 74442, Taiwan; sam.ho@glact.com.tw (H.-H.H.); vic.kuo@glact.com.tw (Y.-W.K.); Shin-Yu.Tsai@glact.com.tw (S.-Y.T.); kenny.chen@glact.com.tw (C.-W.C.); juifen.chen@glact.com.tw (J.-F.C.); 5 Department of Sports Training Science-Athletics, National Taiwan Sport University, Taoyuan City 33301, Taiwan; cychen@ntsu.edu.tw; 6 Department of Medical Laboratory Sciences and Biotechnology, Fooyin University, Kaohsiung City 83102, Taiwan; glycosamine@yahoo.com.tw; 7 Aging and Disease Prevention Research Center, Fooyin University, Kaohsiung City 83102, Taiwan

**Keywords:** OLP-01, *Bifidobacterium longum*, probiotic, exercise performance, athletes

## Abstract

*Bifidobacterium longum subsp. longum* Olympic No. 1 (OLP-01) has been shown in previous animal experiments to improve exercise endurance performance, but this effect has not been confirmed in humans, or more particularly, in athletes. Toward this end, the current study combined OLP-01 supplementation with regular exercise training in well-trained middle- and long-distance runners at the National Taiwan Sport University. The study was designed as a double-blind placebo-controlled experiment. Twenty-one subjects (14 males and seven females aged 20–30 years) were evenly distributed according to total distance (meters) traveled in 12 min to one of the following two groups: a placebo group (seven males and three females) and an OLP-01 (1.5 × 10^10^ colony forming units (CFU)/day) group (seven males and four females). All the participants received placebo or OLP-01 supplements for five consecutive weeks consisting of three weeks of regular training and two weeks of de-training. Before and after the experiment, the participants were tested for 12-min running/walking distance, and body composition, blood/serum, and fecal samples were analyzed. The results showed that OLP-01 significantly increased the change in the 12-min Cooper’s test running distance and the abundance of gut microbiota. Although no significant change in body composition was found, OLP-01 caused no adverse reactions or harm to the participants’ bodies. In summary, OLP-01 can be used as a sports nutrition supplement, especially for athletes, to improve exercise performance.

## 1. Introduction

Middle- and long-distance running is defined as competitions of 800 m to 10,000 m and require both speed and endurance. From a performance optimization perspective, these events are very complex. For example, elite middle- and long-distance runners need to develop aerobic exercise systems similar to those of marathon runners, as well as some mechanical properties of elite sprinters, while possessing world-class anaerobic ability and superb tactical instincts to maintain the fastest possible speed throughout the race [1,2]. In addition to genetics, other factors, such as gender, age, physiological and psychological factors [3,4], training [5], metabolic variables [6], energy utilization [7], and nutritional supplements [8], have important impacts on exercise performance.

Endurance can be defined as the ability to maintain one’s speed or power output for as long as possible [9]. However, endurance performance is related to fatigue, which is a complex physiological phenomenon. It can be divided into central fatigue and peripheral fatigue. The physical and psychological effects during exercise depend on the type, intensity, duration, and energy expenditure of the exercise. They are used to describe the decline in physical function and the actual/perceived difficulties associated with tasks or increased exercise [10]. To cope with long races and high-tension speeds, middle- and long- distance runners need more complex energy metabolism, which is characterized by aerobic metabolism, glycolysis, and adenosine triphosphate-creatine phosphate (ATP-cp), a combined energy metabolism of three energy supply systems [11]. As the distance increases, the type of metabolism gradually changes from a mixed metabolic process based on anaerobic metabolism to a mixed metabolic process based on aerobic metabolism [12]. These energy systems interact with tissues, including muscle types, fibers, and mitochondria, to provide the large amounts of energy required for strenuous exercise. Furthermore, recently-described complex interrelationships between gut microbiota and systemic energy metabolism exert positive performance effects in elite athletes [13].

Exercise training can cause changes in gut microbiota and increase their diversity and abundance [14]. Athletes have a higher short-chain fatty acid (SCFA) metabolism pathway [15], which involves digesting complex carbohydrates and fermentation in the colon. In addition, propionate and acetate are transported through the blood to various organs as a substrate for energy metabolism, particularly to hepatic cells for gluconeogenesis with propionate [16]. Butyric acid is transported to mitochondria and recombined under aerobic conditions to become acetyl-CoA, which enters the Krebs cycle, forms NADH, and then enters the electron transport chain, thereby generating ATP production and CO_2_ [17]. Moreover, gut microbiota can also promote the metabolism and synthesis of secondary bile acid to directly modify mitochondrial biogenesis, inflammation, and intestinal barrier function, and in combination with SCFA can improve energy efficiency and anti-fatigue, thereby enhancing exercise performance [18,19].

In addition to exercise training, the proportion and time of dietary intake had a great influence on athletes′ energy expenditure and exercise performance [20]. Diet is also one of the important factors affecting the gut microbiota and proportion [21]. Dietary or probiotic supplements are the most direct and effective ways to increase the richness and diversity of gut microbiota. Probiotics are considered to be beneficial microorganisms for the host, since they benefit intestinal and physical health [22]. Different strains have different effects, but exercise-related research on them is still in its infancy. Currently, most research on probiotics is focused on improving respiratory function [22] and reducing intestinal discomfort in athletes [23]. In the area of exercise performance, most studies have been based on animal models [24,25], and few studies have confirmed the beneficial effects on the human body [26,27]. One six-week study found that supplementation with *Lactobacillus plantarum* TWK10 can lead to significant improvements in endurance exercise performance and body composition in both male and female humans, and it also reduced fatigue indicators [27]. Therefore, more experiments are needed to confirm the efficacy and mechanism of probiotics to improve sports performance.

*B. longum* OLP-01 was isolated from a weightlifting gold medalist. In previous studies, it was found to significantly increase muscle strength and endurance exercise performance and to reduce the fatigue index of untrained or trained mice [28,29]. Therefore, in the current study, we combined OLP-01 with three weeks of regular training and two weeks of de-training for middle- and long-distance runners at the National Taiwan Sports University to explore of the effects of OLP-01 supplementation on endurance exercise performance and physiological adaptation.

## 2. Materials and Methods

### 2.1. Probiotic

OLP-01, a human strain probiotic derived the *Bifidobacterium longum subsp. Longum*, was isolated from an Olympic gold medalist in the women′s 48 kg weightlifting event. The OLP-01 in the current study was identified by an independent third party, the Food Industry Research and Development Institute (Hsinchu, Taiwan), and prepared and provided by Glac Biotech Co., Ltd. (Tainan, Taiwan) in the form of capsules containing the specified dose. Each OLP-01 capsule contained 5 × 10^9^ colony forming units (CFU). The placebo capsules were indistinguishable in appearance from the OLP-01 capsules. The dosage was three capsules per day, one after each meal.

### 2.2. Participants

Participants included healthy people aged 20 to 30 and well-trained middle- and long-distance runners, but excluded people with high blood pressure, asthma, or skeletal neuromuscular injuries in the upper or lower extremities. Participants were instructed to cooperate with the corresponding training courses and not to consume nutritional supplements, yogurt, Yakult, other probiotic-related products, or antibiotics during the experiment, and they abstained from alcohol consumption for 1 week before the exercise test. The study was approved and reviewed by the Landseed International Hospital Institutional Review Board (Taoyuan, Taiwan; LSHIRB No. 19-005-A2). After the experiment process and content were explained in detail, all volunteers provided written informed consent before participating.

### 2.3. Experimental Design

**Figure 1:**
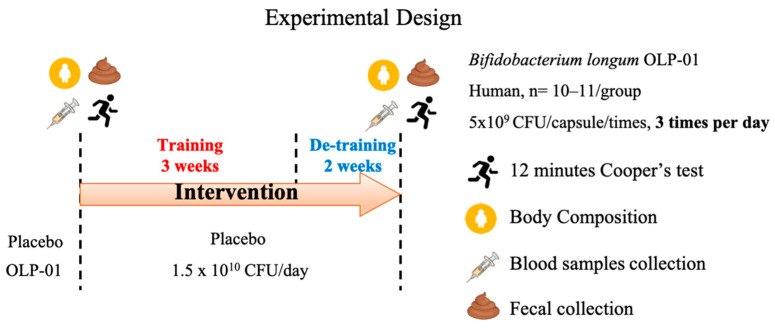
Experimental design. We used a double-blind test in which volunteers (21 subjects; 14 males and seven females) were assigned to two groups: a placebo group and an OLP-01 (1.5 × 1010 colony forming units (CFU)/day) group. They completed a five-week intervention consisting of three weeks of regular training and two weeks of de-training, and their physical fitness, physiological adaptations, and fecal samples were analyzed before and after the experimental intervention.

Twenty-one (14 males and seven females aged 20–30 years) well-trained middle- and long-distance runners at the National Taiwan Sports University were recruited. We used a double-blind test in which the participants were evenly distributed according to total distance (meters) traveled in 12 min to one of two groups: a placebo group (seven males and three females) and an OLP-01 (1.5×10^10^ CFU/day) group (seven males and four females). The experimental procedure is presented in Figure 1. All the participants received placebo or OLP-01 supplements for five consecutive weeks, which included three weeks of regular training and two weeks of de-training. Before and after the experiment, the participants were tested for 12-min running/walking distance, and their body composition, blood/serum, and fecal samples were analyzed. During the experiment, all the subjects cooperated with the team for work and rest. The team dietitian specified the diet and provided the same meal to ensure the consistency of the diet. The basic demographics and characteristics of the subjects are listed in Table 1.

### 2.4. The 12-min Cooper Running/Walking Test

The 12-min Cooper running/walking test was used as a preliminary and simple method to assess aerobic endurance and physical fitness [30]. A standard sports field of 400 m was marked every 10 m. The time was recorded from the start of running, and the distance traveled was recorded every 3 min (3rd, 6th, 9th, and 12th min).

### 2.5. Body Composition

Body composition was measured according to the multi-frequency principle with the bioelectrical impedance analyzer (BIA) of the InBody 570 (In-body, Seoul, Korea), which provides frequency screenings of 1, 5, 50, 260, 500, and 1000 kHz within 60 s. After their palms and soles were cleaned, the subjects stood upright on the electrodes of the instrument, held the sensing handle in both hands with the arms away from the body at a 30° angle, and refrained from speaking or moving during the measurement period [26].

### 2.6. Blood Routine and Serum Biochemical Analysis

To understand the health status of the subjects and whether they were adversely affected by the OLP-01 supplemented training, we collected blood with an arm venous catheter for analysis at the beginning and end of the experiment. The physiological adaptations and clinical biochemistry of the blood serum were assessed with an autoanalyzer (Hitachi 7060, Tokyo, Japan) for lactate, ammonia, creatinine kinase (CK), glucose, aspartate transaminase (AST), alanine aminotransferase (ALT), albumin, total protein (TP), total cholesterol (TC), triglyceride (TG), high-density lipid (HDL), low-density lipid (LDL), blood urea nitrogen (BUN), creatinine, and uric acid (UA) levels.

### 2.7. Bacterial DNA Extraction and 16S rRNA Sequencing

Before the start and the designated end point, fecal specimens were collected from all subjects, and the DNA/RNA Shield™ reagent was used in a fecal collection tube (Zymo Research Corp, Irvine, CA, USA). Each collection tube (with a spoon attached to the lid) was pre-filled with DNA/RNA Shield™ (9 mL). Bacterial DNA was extracted by the cetyltrimethylammonium bromide/sodium dodecyl sulfate (CTAB/SDS) method, and the obtained nucleic acids (DNA and RNA) were stored at −80 °C for subsequent analysis. DNA purity was determined by the ratio of OD 260 to OD 280 in the range of 1.8–2.0. Specific primers 341F (F, forward primer; 5′-CCTAYGGGRBGCASCAG-3′) and 806R (R, reverse primer; 5′-GGACTACNNGGGTATCTAAT-3′) were used to amplify the highly variable V3-V4 regions of the bacterial 16S rRNA gene by PCR Zone. A sample preparation kit (Illumina, San Diego, CA, USA) without TruSeq DNA PCR was used to construct a double-ended library (each sample insertion size was 450–470 bp). The amplified DNA sizing was checked by TapeStation (Agilent Technologies, Santa Clara, CA, USA). High-throughput sequencing was performed on the Illumina HiSeq2500 platform. The sequences thus generated were filtered to obtain valid reads. Total reads were merged, low-quality and chimera sequence sequences were removed, and the clustered operational taxonomic unit (OTU) had 97% similarity to the Greengenes database. All OTU sequences and diversity analysis were performed with the CLC Microbial Genomics Module (Qiagen, Hilden, Germany), basespace (Illumina, San Diego, CA, USA) and Graphpad prism 7 (Graphpad Software, San Diego, CA, USA). A *p*-value of less than 0.05 was considered statistically significant.

### 2.8. Statistical Analysis

All the data are expressed as mean ± SEM. Statistical analyses were performed in SAS 9.0 (SAS Inst., Cary, NC, USA). Multi-group comparisons were analyzed by one-way analysis of variance (ANOVA), and within-group differences (before vs. after OLP-01 supplementation), by paired Student’s *t*-test. Statistical significance was set at *p* < 0.05.

## 3. Results

### 3.1. Effect of OLP-01 Supplementation on Distance in 12-min Cooper Running/Walking Test

**Figure 2:**
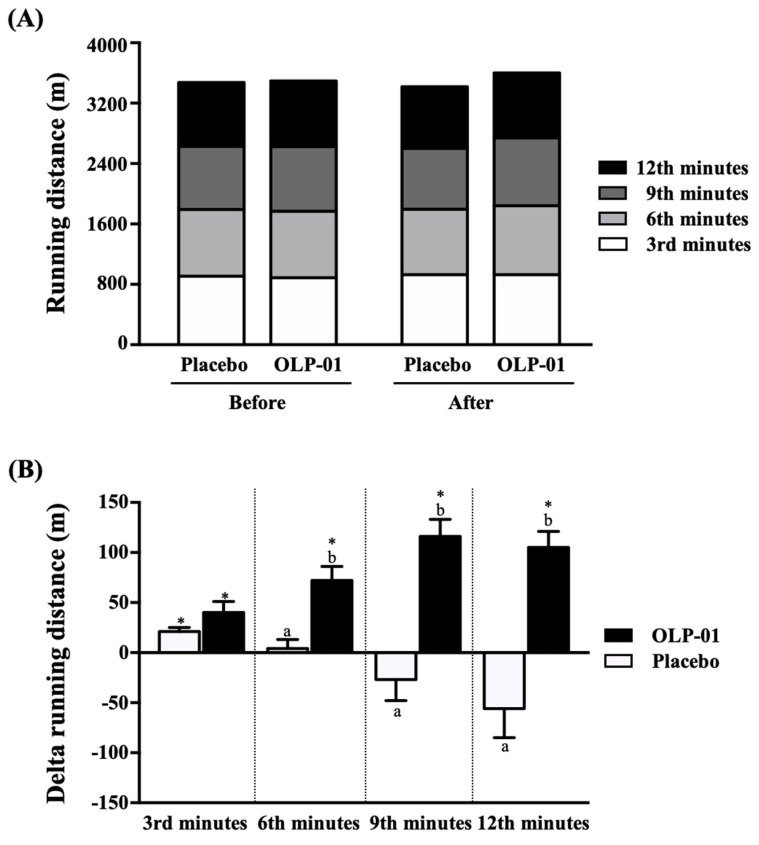
Effects of OLP-01 supplementation combined with three weeks of regular training and two weeks of de-training on (**A**) running distance and (**B**) change in running distance. Data are presented as mean ± SD. Different superscript letters (a, b) indicate significant difference at *p* < 0.05, and pre- and post-administration effects were statistically analyzed using a paired Student’s *t*-test, * *p* < 0.05, at each time point (3rd, 6th, 9th, and 12th min).

As shown in Figure 2A, before the intervention, the placebo group and the OLP-01 supplementary group achieved distances of 907 ± 26 and 888 ± 21 m, 1791 ± 52 and 1768 ± 39 m, 2629 ± 74 and 2625 ± 65 m, and 3475 ± 101 and 3496 ± 84 m at the 3rd, 6th, 9th, and 12th min, respectively. There were no significant differences between them. After five consecutive weeks of OLP-01 intervention combined with three weeks of regular training and two weeks of de-training, the placebo group and the OLP-01 supplementary group achieved distances of 927 ± 28 and 928 ± 28 m, 1795 ± 57 and 1840 ± 48 m, 2602 ± 83 and 2740 ± 74 m, and 3419 ± 112 and 3602 ± 86 m at the 3rd, 6th, 9th, and 12th min, respectively. There were no significant differences between them.

Despite the lack of significant differences in running distance before or after supplementation between the two groups, we further analyzed the differences between post-test and pre-test to compare improvements in running distance. As shown in Figure 2B, the placebo group and the OLP-01 supplementary group exhibited changes in running distance of 21 ± 4 and 40 ± 11 m, 4 ± 9 and 72 ± 14 m, −27 ± 21 and 116 ± 17 m, and −56 ± 29 and 105 ± 16 m at the 3rd, 6th, 9th, and 12th min, respectively. Compared with the placebo group, the improvements in the OLP-01 group were significantly greater at the 6th min (*p* = 0.0014), 9th min (*p* = 0.0001) and 12th min (*p* = 0.0001).

In addition, relative to the pre-test, the placebo group only exhibited improvement in running distance in the post-test at the 3rd minute (*p* = 0.0051), and there were no significant differences at the other time points. In the OLP-01 group, running distances in the post-test were significantly greater at the 3rd (*p* = 0.0051), 6th (*p* = 0.0004), 9th (*p* < 0.0001), and 12th (*p* < 0.0001) minute.

### 3.2. Effect of OLP-01 Supplementation on Body Composition

Before and after the experimental intervention, body composition analysis was performed. To ensure the consistency of the experiment, the same machine was used for the same time and under the same conditions. As shown in Table 2, there were no significant differences in body weight, body mass index (BMI), or body fat percentage between the placebo and OLP-01 groups before or after the experimental intervention. Only the placebo group exhibited a significant reduction in muscle mass (*p* = 0.0370) after the intervention.

### 3.3. Effect of OLP-01 Supplementation on Blood Biochemistry

Blood was collected from all subjects before and after the intervention for basic value testing and safety assessment. Liver function (AST, aspartate transaminase; ALT, alanine aminotransferase; ALB, albumin; TP, total protein), kidney function (BUN, blood urea nitrogen; CREA, creatinine; UA, uric acid), blood lipid (TC, total cholesterol; TG, triglyceride; HDL, high-density lipid; LDL, low-density lipid), Lactate, ammonia (NH_3_), glucose, and creatine kinase (CK) were tested. There were no significant differences between groups in these indexes (Table 3). 

### 3.4. Effect of OLP-01 Supplementation on Complete Blood Count Profiles

Blood samples for blood count profiles were collected and analyzed at the same times as those for the blood biochemistry, and the complete blood counts of the volunteers before and after OLP-01 administration were obtained. As shown in Table 4, no significant differences in WBC, neutrophil, lymphocyte, monocyte, eosinophil, basophil, or platelet indices were detected between or within groups.

The ratio of neutrophil count to lymphocyte count (NLR) and the ratio of platelet count to lymphocyte count (PLR) were used as markers of exercise-induced systemic inflammation. NLR and PLR were calculated according to the specified reference index, and no significant differences between groups were found.

### 3.5. Effect of OLP-01 Supplementation on Gut Microbiota

**Figure 3:**
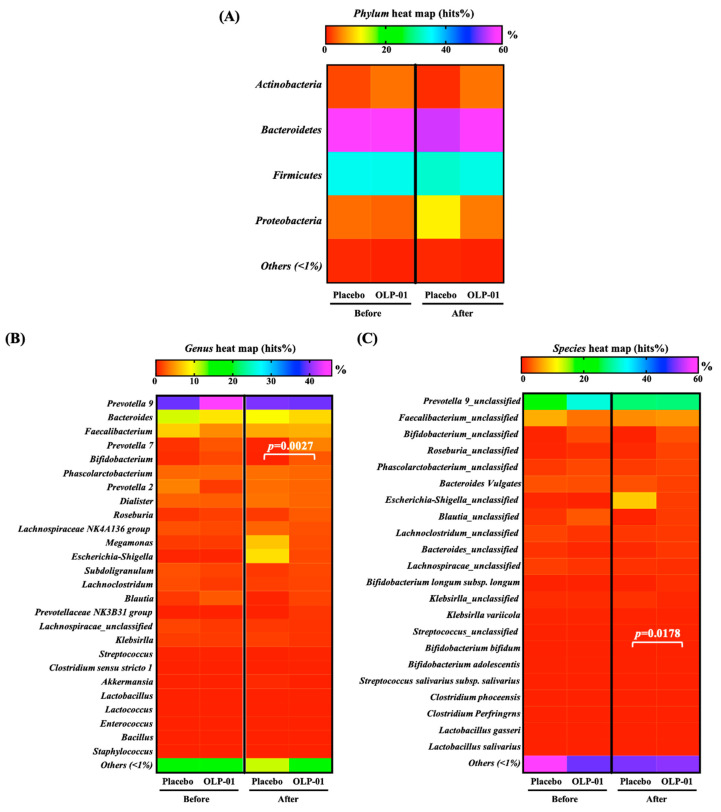
Gut microbiota heat map (hits%) of (**A**) *Phylum*, (**B**) *Genus*, and (**C**) *Species*. Data are expressed as mean ± SEM and indicate significant difference at *p* < 0.05.

We used the 16S rRNA gene to analyze the composition of gut microbiota. At the *Phylum* level, there were no significant differences in *Actinobacteria, Bacteroidetes*, *Firmicutes*, or *Proteobacteria* between the placebo and OLP-01 groups before the experimental intervention (Figure 3A). Interestingly, after five weeks of supplementation with OLP-01, the populations of *Actinobacteria* and *Firmicutes* were more abundant in the OLP-01 group than in the control group, and *Proteobacteria* was less abundant in the OLP-01 group than in the control group.

At the *Genus* level (Figure 3B), no significant differences between placebo and OLP-01 groups existed before the experimental intervention. After five weeks of supplementation with OLP-01, *Bifidobacterium* was significantly richer in the OLP-01 group than in the control group (*p* = 0.0027). Especially, the *Lactobacillus* count also increased nine-fold after supplementation with OLP-01.

After five weeks of supplementation with OLP-01, at the *Species* level, *Bifidobacterium longum* subsp. *longum* in the placebo and OLP-01 groups were 0.11% and 0.95%, respectively (Figure 3C). In the OLP-01 group, *Bifidobacterium longum* subsp. *longum* significantly increased by 8.63-fold (*p* = 0.0178). It was confirmed that OLP-01 colonized the human intestine, thereby increasing the number of *B. longum* subsp. *longum*. In addition, although the amounts of common strains and pathogenic strains in the OLP-01 group were not significantly different from those in the placebo group. *Escherichia-Shigella* in the gut in the OLP-01 group decreased by 81.03%. Based on the above, the five-week OLP-01 supplementation significantly improved the *B. longum* subsp *longum* species, increased the abundance of other probiotics, and reduced the numbers of certain pathogenic bacteria in the participants.

## 4. Discussion

The probiotic OLP-01 isolated from the 2008 Olympic women’s 48 kg weightlifting gold medalist’s gut microbiota has been proven in past animal experiments to effectively improve exercise endurance performance and reduce fatigue indicators [28,29]. In this study, after the well-trained middle- and long-distance runners were provided OLP-01 probiotic in combination with regular training, we also found that it could effectively colonize the human intestine, thereby maintaining and promoting sports endurance performance. It had neither beneficial effects on body composition nor adverse effects on the human body.

To avoid excessive burden and fatigue in athletes, which would affect daily training and life, we used the endurance test method of daily athletic training, the 12-min Cooper running/walking test, which can be used as a detection method to estimate the maximum oxygen uptake and exercise endurance indicators [31], considering the distance traveled per unit time and the total distance at the end time point as the speed and endurance performance indicators. Since the subjects were all well-trained middle- and long-distance runners, there were no significant differences in running distance between the two groups (Figure 2A). At present, opinions vary on the efficacy of probiotics in improving exercise performance, especially in trained athletes. Previous studies had shown that 14 weeks of supplementation with more than 14 types of probiotics did not affect VO_2max_ or maximum performance [32]; however, positive results were reported in endurance-trained men. Four weeks of supplementation with multiple strains of probiotics (45 billion CFU of *Lactobacillus*, *Bifidobacterium*, and *Streptococcus* strains) increased the fatigued running time of athletes in hot environments [33]. In addition, 30 endurance athletes were given a yogurt drink containing either *Streptococcus thermophilus* or *Lactobacillus delbrueckii ssp. bulgaricus*, or no probiotics, over 30 days during intense aerobic training. The results indicated a significant increase in VO_2max_ and aerobic power in the Cooper aerobic test [34]. The effects of probiotics on improving exercise performance may be related to the supplemented strain, dosage and time. Supplementing *Bifidobacterium longum* 35,624 daily during a six-week exercise training phase does not directly affect exercise performance. However, the probiotic group reported higher exercise recovery during the last two weeks of the off-season training program [35]. That finding was similar to ours; after three weeks of intensive training and cessation of training for two weeks, the running distance of the OLP-01 group not only was maintained but increased relative to their performances before OLP-01 intervention, and the change was also significantly greater than that of the placebo group (Figure 2B). In this trial, the only component of the placebo was maltodextrin, which is a legal food additive and has been used as a placebo in many experiments. In addition, it is also used as an additive for probiotic encapsulation. It has the ability to increase weight after digestion in the gastrointestinal tract and maintain the viability of the strain [36]. In addition, previous studies focused on the use of maltodextrin as a carbohydrate supplement as an energy source, and found that it does not affect sports performance [37].

Both lactic acid and blood ammonia are used to assess energy expenditure and sports fatigue. They increase with increments of exercise intensity and time and begin to decline after rest [38]. During competition, serum UA (a final waste product of protein, amino acids, and DNA), CK (a catalyzing enzyme of creatine phosphorylation in the phosphagen energy system), and LDH (a rate-limiting enzyme that regulates the reaction of pyruvate to lactic acid in the TCA terminal glycolysis system) also increase according to energy and muscle damage [39]. AST and ALT are aminotransferases; both increased on day 2 and were prolonged after the race, but ALT stayed within the normal range. AST is located in skeletal muscle, red blood cells, the heart, and the liver, while ALT leaks from damaged liver cells and skeletal muscle. In addition, during and after competition, TG decreases because TG is mobilized from viscera and subcutaneous fat tissue together with TG in LDL and is broken down into FFA by lipase [40,41]. In the current study, before the exercise tests before and after the intervention, blood samples were collected. The purpose was to understand whether OLP-01 supplementation would cause adverse reactions or harm to the subjects. All blood biochemistry values were in normal ranges, and no significant differences between the placebo and OLP-01 groups were found before or after the five-week intervention (Table 3).

Changes in the composition of human microbes may actually promote chronic inflammation and catabolic metabolism, ultimately regulating the reduction in muscle size, impaired muscle function and poor clinical effectiveness [42]. In this possible gut-muscle axis, age-related intestinal mucosal barrier dysfunction may play a central role [43]. The aging microbiota leads to a reduction in SCFA production, which can promote insulin resistance, reduce mitochondrial fatty acid oxidation and lead to increased fatty acid deposition in muscles. This phenomenon will lead to a decrease in muscle strength and quality, and further promote insulin resistance, thereby promoting the occurrence of a vicious cycle, which ultimately leads to sarcopenia and physical weakness [44]. However, metabolites derived from the gut microbiota may not only include SCFA in promoting skeletal muscle anabolism, phenolic compounds produced by the microbiota can also increase glucose uptake in muscle fibers, induce anabolic reactions, and thereby increase muscle mass [45]. Long-term exercise is a physiological stress that promotes excessive inflammation after an anti-inflammatory compensatory response, thereby reducing exercise performance [46]. Furthermore, acute muscle inflammation caused by intense contraction during exercise may lead to leukocyte infiltration and increased levels of inflammatory cytokines such as tumor necrosis factor-alpha and interleukin-6 [47]. In order to counteract these inflammatory reactions caused by vigorous exercise, the intestinal flora and the SCFA metabolites it produces can regulate neutrophil function and migration, reduce colonic mucosal permeability, inhibit inflammatory cytokines, and control the cells’ Redox environment. These anti-inflammatory effects of the gut microbiota may help to enhance muscle renewal ability and adaptability, help delay fatigue symptoms, and improve endurance performance [48]. Previous studies have confirmed that the values of NLR and PLR can be used as markers of systemic inflammation [49]. The subjects were asked not to exercise for three days before blood collection, thus, in these results, all values were within the normal ranges, and no inflammation occurred (Table 4).

Athletes with long-term regular exercise training have higher gut microbiota diversity [17]. Among athletes, those of long-term endurance events have relatively rich populations of *Prevotella*. Moreover, this study also found a positive correlation of abundance and exercise. Athletes who train for more than 16 h per week have a higher proportion of *Prevotella* [50], which seems to be related to the fact that endurance athletes need large quantities of carbohydrates for energy and consume more dietary fiber or carbohydrates [51]. This phenomenon was also verified in the current study. Among our subjects, the most abundant comrade-in-arms was *Prevotella* (Figure 3B). In our previous study, it was confirmed that OLP-01 supplementation in mice can increase the proportion of *B*. *longum* in the gut microbiota, as well as muscle strength and endurance, and it can also reduce fatigue biochemical values after exercise. Although the mechanism in the human body in this study has yet to be confirmed, the above results showed that the five-week OLP-01 supplementation significantly improved the population of *B. longum* subsp. *longum*, increased the abundance of other probiotics, and reduced the populations of certain pathogenic bacteria. Perhaps increasing the duration of OLP-01 supplementation in future clinical trials will produce more significantly different bacterial phase changes and further exciting results.

The limitations of the study included: first, in order to minimize the changes in training methods, lifestyle, and diet, middle- and long-distance runners were recruited from a single university; thus, the number of participants was small and the ratio of men and women could not be equal. Second, in order not to affect the athlete′s daily training and competition preparation, all tests and interventions were coordinated with the training schedule of the middle- and long-distance running team; thus, it was impossible to intervene for a long time or study the effect of the changes during exercise or recovery.

## 5. Conclusions

This study, unlike previous studies on athletes, confirmed that OLP-01 supplementation combined with regular exercise for three weeks and rest for two weeks can still effectively improve endurance exercise performance and increase *B*. *longum* in well-trained middle- and long-distance runners. The proportion of bacterial genera of other benefic bacteria also increased and the number of certain pathogenic bacteria decreased. In addition, OLP-01 causes no adverse reactions or harm to the human body.

## Figures and Tables

**Table 1:** Basic information data of the subjects.

Characteristic	Placebo	OLP-01
Age (y)	21.2 ± 0.4	21.6 ± 0.7
Height (cm)	168.7 ± 1.5	169.5 ± 2.3
Weight (kg)	57.1 ± 1.9	56.4 ± 1.1
BMI (kg/m^2^)	20.0 ± 0.4	19.7 ± 0.3

Data are expressed as mean ± SEM. There were no significant differences in the basic information data between the two groups.

**Table 2:** Body Composition of subjects.

Characteristics	Before Intervention		After Intervention		Change (After-Before)	
	Placebo	OLP-01	Placebo	OLP-01	Placebo	OLP-01
**BW (kg)**	57.1 ± 1.9	56.4 ± 1.1	56.7 ± 2.0	56.3 ± 1.2	−0.5 ± 0.4	−0.1 ± 0.3
**BMI (kg/cm^2^)**	20.0 ± 0.4	19.7 ± 0.3	19.9 ± 0.5	19.6 ± 0.3	−0.1 ± 0.2	0.0 ± 0.1
**LBM (kg)**	27.8 ± 1.3	27.9 ± 1.2	27.6 ± 1.3	27.8 ± 1.2	−0.1 ± 0.2 *	−0.1 ± 0.2
**FBM (%)**	13.1 ± 1.4	12.1 ± 2.1	13.3 ± 1.3	12.3 ± 2.0	0.2 ± 0.4	0.2 ± 0.2

Data are expressed as mean ± SEM. There were no significant differences between the two groups. Before and after administration effects were statistically analyzed by paired Student’s *t*-test. * *p* < 0.05. BW, body weight BMI, body mass index; LBM, lean body mass; FBM, fat body mass.

**Table 3:** Safety Assessment of Blood Biochemical of Subjects.

Characteristics	Before Intervention		After Intervention	
	Placebo	OLP-01	Placebo	OLP-01
Lactate (mmol/L)	2.41 ± 0.10	2.41 ± 0.12	1.86 ± 0.12	2.07 ± 0.07
NH_3_ (μmol/L)	117 ± 6	119 ± 4	88 ± 13	88 ± 12
CK (U/L)	187 ± 25	183 ± 21	191 ± 21	179 ± 16
Glucose (mg/dL)	89 ± 2	85 ± 2	91 ± 2	89 ± 2
AST (U/L)	26 ± 2	23 ± 2	24 ± 3	24 ± 3
ALT (U/L)	22 ± 3	21 ± 1	19 ± 2	18 ± 1
ALB (mg/dL)	4.9 ± 0.1	4.9 ± 0.1	4.9 ± 0.1	4.9 ± 0.1
TC (mg/dL)	168 ± 8	185 ± 1	163 ± 7	169 ± 1
TG (mg/dL)	77 ± 7	77 ± 5	71 ± 6	69 ± 4
HDL (mg/dL)	67.4 ± 2.6	67.2 ± 3.0	63.8 ± 2.2	70.3 ± 3.1
LDL (mg/dL)	86.5 ± 5.1	93.6 ± 3.6	88.6 ± 4.9	81.7 ± 3.0
BUN (mg/dL)	16.5 ± 0.9	16.3 ± 0.8	16.2 ± 0.7	15.6 ± 0.8
CREA (mg/dL)	1.09 ± 0.04	1.10 ± 0.03	1.09 ± 0.04	1.09 ± 0.02
UA (mg/dL)	5.7 ± 0.4	5.1 ± 0.4	6.3 ± 0.6	6.0 ± 0.4
TP (mg/dL)	6.9 ± 0.1	6.9 ± 0.2	7.0 ± 0.2	7.0 ± 0.1

Data are expressed as mean ± SEM. There were no significant differences between the two groups. NH_3_, blood ammonia, CK, creatine kinase; AST, aspartate transaminase; ALT, alanine aminotransferase; ALB, albumin; TC, total cholesterol; TG, triglyceride; HDL, high-density lipid; LDL, low-density lipid; BUN, blood urea nitrogen; CREA, creatinine; UA, uric acid; TP, total protein.

**Table 4:** Effects of OLP-01 on Complete Blood Count Profiles.

Characteristics	Before Intervention		After Intervention	
	Placebo	OLP-01	Placebo	OLP-01
WBC (cells/mcL)	7089 ± 471	7071 ± 320	7083 ± 487	7106 ± 456
Neutrophils (%)	51.7 ± 2.4	53.0 ± 2.8	51.9 ± 2.1	55.0 ± 2.4
Lymphocytes (%)	39.2 ± 2.2	36.7 ± 2.6	39.1 ± 2.2	35.3 ± 2.0
Monocytes (%)	5.5 ± 0.3	5.8 ± 0.4	5.8 ± 0.2	6.0 ± 0.3
Eosinophil (%)	2.9 ± 0.5	3.8 ± 1.0	2.4 ± 0.6	3.1 ± 0.5
Basophil (%)	0.7 ± 0.2	0.7 ± 0.1	0.8 ± 0.1	0.7 ± 0.1
Platelet (10^3^/mcL)	252 ± 13	273 ± 15	271 ± 13	267 ± 11
NLR	1.39 ± 0.15	1.60 ± 0.25	1.40 ± 0.14	1.65 ± 0.17
PLR	96 ± 8	112 ± 10	102 ± 7	115 ± 11

Data are expressed as mean ± SEM. No significant differences between groups were found. NLR, ratio of neutrophil count to lymphocyte count; PLR, ratio of platelet count to lymphocyte count.
